# NMR structure and function of *Helicoverpa armigera* sterol carrier protein-2, an important insecticidal target from the cotton bollworm

**DOI:** 10.1038/srep18186

**Published:** 2015-12-10

**Authors:** Haihao Ma, Yuemin Ma, Xuehui Liu, David H. Dyer, Pingyong Xu, Kaiyu Liu, Que Lan, Huazhu Hong, Jianxin Peng, Rong Peng

**Affiliations:** 1 School of Life Sciences, Central China Normal University, Wuhan, P.R.China; 2 National Laboratory of Biomacromolecules, Institute of Biophysics, Chinese Academy of Sciences, Beijing, P.R.China; 3 Department of Biochemistry and Entomology, College of Agricultural and Life Sciences, University of Wisconsin-Madison, Madison, United States of America; 4 School of Life Sciences, Wuhan Institute of Bioengineering, Wuhan, P.R.China

## Abstract

The cotton bollworm, *Helicoverpa armigera*, has developed strong resistance to many insecticides. Sterol Carrier Protein-2 (SCP-2) is an important non-specific lipid transfer protein in insects and appears to be a potential new target. In order to elucidate the structure and function of *Helicoverpa armigera* SCP-2 (HaSCP-2), NMR spectroscopy, docking simulations, mutagenesis and bioassays were performed. HaSCP-2 composed of five α-helices and four stranded β-sheets. The folds of α-helices and β-sheets interacted together to form a hydrophobic cavity with putative entrance and exit openings, which served as a tunnel for accommodating and transporting of lipids. Several sterols and fatty acids could interact with HaSCP-2 via important hydrophobic sites, which could be potential targets for insecticides. Mutagenesis experiments indicated Y51, F53, F89, F110, I117 and Q131 may be the key functional sites. HaSCP-2 showed high cholesterol binding activity and SCP-2 inhibitors (SCPIs) could inhibit the biological activity of HaSCP-2. SCPI-treated larvae at young stage showed a significant decrease of cholesterol uptake *in vivo*. Our study describes for the first time a NMR structure of SCP-2 in lepidopteran *H. armigera* and reveals its important function in cholesterol uptake, which facilitates the screening of effective insecticides targeting the insect cholesterol metabolism.

The cotton bollworm, *Helicoverpa armigera* (Hubner) (Lepidoptera: Noctuidae) causes serious crop damage every year all over the world and poses a great threat to the economics of global agricultural production. It feeds on diverse economically important crops, including soybeans, cotton, sorghum, corn, sunflower, peanuts, beans, tomatoes and peppers^1^. Many management strategies have been proposed to control the *H. armigera* in recent years, while as the use of conventional pesticides is still considered to be the fast and effective way^2^. *H. armigera* has developed strong resistance to many insecticides^1^,^2^. There is an urgent need to seek safer insecticides with new modes of action to effectively control the cotton bollworm.

It is well known that cholesterol is an essential component of cell membranes and a starting intermediate compound from which an insect makes steroid hormones, bile acids and vitamin D^3^,^4^. It is intriguing that different from vertebrates, insects are unable to synthesize cholesterol by themselves due to a lack of several key enzymes in the *de novo* cholesterol synthetic pathway^3^,^4^,^5^,^6^. Insects must rely on their host plants to obtain the cholesterol exogenously, which is essential to ensure normal growth, development and reproduction^7^,^8^,^9^. Therefore, the unique pathway of uptake, transfer and accumulating of cholesterol in the body are physiologically critical for insects. Many studies have demonstrated that sterol carrier protein 2 (SCP-2), a non-specific lipid transfer protein, is involved in the absorption and transportation of steroid or lipids in insects^10^,^11^,^12^,^13^,^14^,^15^,^16^,^17^. SCP-2 belongs to the SCP-2 gene family including SCP-X, SCP-2, 17β-hydroxysteroid dehydrogenase IV, stomatin, UNC-24, and Metallo-β-lactomase and is identified in many species including vertebrates, insects, plants, yeast, bacteria and fungi^18^,^19^,^20^. All the members in this family share a homologous SCP-2 domain, which is generally located at the C-terminus. Moreover, the SCP-2 domain exhibits a high sequence identity to other SCP-2′s from many different organisms, which implies the SCP-2 family may have a conserved structure and function during the long period of evolution.

Sterol carrier proteins have been mainly implicated in a wide array of cholesterol/lipid related functions in vertebrates and insects^21^,^22^,^23^. Recent studies have demonstrated that SCP-2 has cholesterol/lipid binding activities^21^,^22^,^23^,^24^. SCP-2 can bind to cholesterol, palmitic acid, fatty acyl-CoA, acidic phospholipids and bile salts^25^,^26^,^27^,^28^,^29^,^30^,^31^. The binding affinity of SCP-2 to cholesterol is the strongest among the lipids.

To date, the knowledge of the SCP-2 domain protein structure is limited and is primarily focused in vertebrates^32^,^33^,^34^,^35^,^36^. In insects, where SCP-2 is crucial for their life cycles, few studies on SCP-2 structure are reported. The three-dimensional structures of *Aedes aegypti* SCP-2 proteins from dipteran mosquitoes are determined by X-ray diffraction and NMR spectroscopy, respectively^25^,^28^,^29^,^37^.

In this paper, in an effort to understand the structure and function of lepidopteran SCP-2, NMR spectroscopy were carried out to determine the three-dimensional structure of cotton bollworm, *H. armigera* SCP-2 (HaSCP-2) for the first time. Meanwhile, mutagenesis, molecular docking and *in vivo* bioassays were performed to detect the ligand binding affinity of HaSCP-2 and SCP-2 inhibitors. The results from NMR analysis of the HaSCP-2 functional domain, the computational molecular docking and *in vivo* bioassays revealed the important function of HaSCP-2 that serves as a sterol/lipid transporter in the insect. Therefore, HaSCP-2 can be an important insecticidal target for controlling *H. armigera*. The exploration of HaSCP-2 NMR structure and function not only provides insight into the mechanism of ligand binding function of this SCP-2 protein family, but also can eventually facilitate the computer aided designing of insecticides with new modes of action, targeting the cholesterol and lipid metabolism.

## Results

### Expression and identification of HaSCP-2

**Figure 1:**
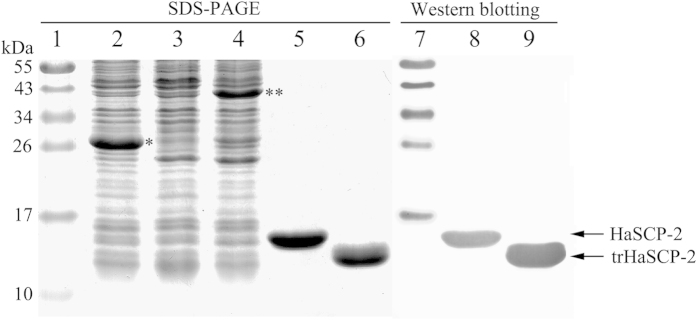
The expression of recombinant GST-HaSCP-2 in *E. coli* and western blotting analysis. In the experiment of HaSCP-2 expression, the supernatant of *E. coli* lysate was used for SDS-PAGE and western blotting analysis. Protein samples were loaded on 15% SDS-PAGE. Lane 1: molecular weight standards (kDa); Lane 2: the total protein of *E. coli* BL21(DE3) carrying plasmid pGEX-KG with IPTG induction, *indicated the GST protein with a molecular weight of 26.5 kDa; Lane 3: the total protein of *E. coli* BL21 (DE3) carrying plasmid of pGEX-KG-HaSCP-2 without IPTG induction; Lane 4: total protein of *E. coli* BL21(DE3) carrying plasmid of pGEX-KG-HaSCP-2 with IPTG induction, **indicated the GST-HaSCP-2 fusion protein with a molecular weight of about 42 kDa; Line5: purified HaSCP-2 protein with molecular weight of about 16 kDa; Line6: purified truncated HaSCP-2 (trHaSCP-2) protein with molecular weight of about 14 kDa; For western blotting detection with anti-AeSCP-2 antibodies (anti-*Aedes aegypti* antibodies), Lane 7: the same molecular weight standards as Lane 1; Lane 8: purified HaSCP-2 protein was dected; Lane 9: purified truncated trHaSCP-2 protein was detected.

A recombinant *H. armigera* SCP-2 (HaSCP-2) protein fused with a GST-tag of 42442 Da was successfully expressed upon induction with IPTG in *E. coli* (Fig. 1). The protein was identified by SDS-PAGE and western blotting as shown in Fig. 1. The expressed fusion protein was primarily purified by GST resin affinity column and Thrombin digestion to remove the GST-tag. Then the HaSCP-2 protein (without GST-tag) with a molecular weight of 16293 Da was purified by using anion exchange chromatography and gel filtration purification. The purified HaSCP-2 protein with a molecular weight of ~16 kDa and truncated protein (trHaSCP-2, described in the following text) with a molecular weight of ~14 kDa were both detected by using anti-*Aedes aegpty*SCP-2 antibodies (Fig. 1). These results suggested that a recombinant HaSCP-2 protein and trHaSCP-2 were obtained successfully in *E. coli*.

### The truncation of HaSCP-2 for NMR

**Figure 2:**
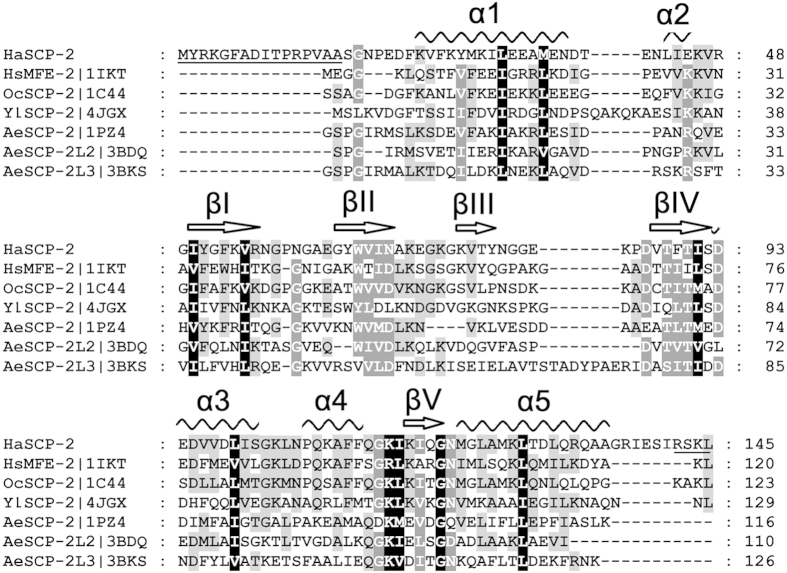
The amino acid sequence alignment analysis of SCP-2s. The alignment was prepared with the program ClustalX2 (http://www.clustal.org) and processed by GeneDoc software (www.psc.edu/biomed/genedoc). The amino acids underlined were absent in the protein sample (truncated HaSCP-2) for the NMR spectometry analysis.

The deduced SCP-2 domain of *H. armigera* HaSCP-2 was compared with that of human, rabbit, mosquito, and yeast by using the ClustalX 2.0 online program (ftp://ftp.ebi.ac.uk/pub/software/clustalw2) (Fig. 2). It was found that compared with the reported SCP-2 domain structures from human, rabbit, mosquito, and yeast (PDB databases, www.rcsb.org/pdb/home/home.do), deduced *H. armigera* SCP-2 domain exhibited an extra sequence about 16 amino acid residues at N-terminus (Fig. 2 HaSCP-2 N-terminus: MYRKGFADITPRPVAA ). It was reported that an N-terminal fragment about 16–20 amino acids was released from the pro-SCP-2 upon rapid post-translational cleavage of the pro-SCP-2^20^. The N-terminal amino acids are mainly responsible for intracellular targeting of SCP-2 to peroxisomes^24^. And it was demonstrated that the N-terminal truncation of 16 amino acids did not affect the binding ability of HaSCP-2 to NBD-cholesterol (as showed in the following NBD-cholesterol binding assay experiments). Therefore, the sequence of 16 amino acids residues was primarily deleted from the N-terminal end of HaSCP-2 protein for the following NMR experiment. On the other hand, it was interestingly observed that there was four amino acids degradation at the C-terminus of the deduced HaSCP-2 domain during the molecular weight analysis of MALDI-TOF Mass Spectrometry (data not shown) after purifying recombinant protein. It was reported that SKL sequence in C-terminal was peroxisomal targeting signal and it was cleaved in mature SCP-2 protein^20^, which suggested that it may not be involved in the binding function of SCP-2 to lipids. Therefore, the RSKL sequence at C-terminus was deleted for the NMR analysis of HaSCP-2 domain. It was also found that C-terminal truncation does not affect the important function of HaSCP-2 in binding with cholesterol (as showed in the following NBD-cholesterol binding assay experiments). The truncated HaSCP-2 with both N-terminus and C-terminus truncation has the similar binding activity to that of deduced HaSCP-2. Finally, a truncated HaSCP-2 protein with both the N-terminal and the C-terminal deletions was applied to the NMR analysis.

### The three-dimensional structure of HaSCP-2 by NMR

**Figure 3:**
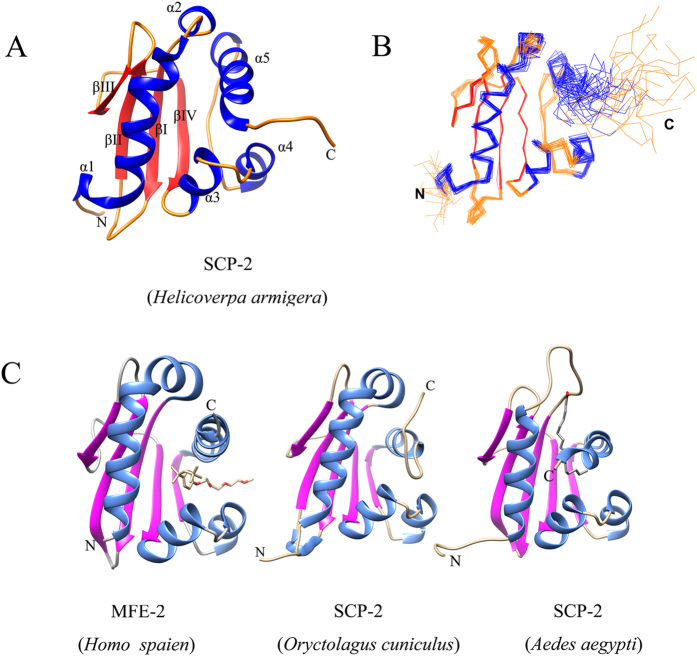
NMR structure of *Helicoverpa armigera* SCP-2. (**A**) The stereo ribbon representations of *Helicoverpa armigera* SCP-2 three-dimension structure. The α-helices are labeled with number “1–5” and stranded β-sheets are labeled with number “I–IV”. N- and C-termini are labeled as “N” and “C” , respectively. (**B**) Superposition of the 20 conformers with the lowest energy in NMR spectometry. (**C**) Tertiary structures of sterol carrier protein from human, rabbit and mosquito.

By NMR spectroscopy, a three-dimensional structure of *H. armigera* SCP-2 was determined (Fig. 3A,B). In NMR analysis, the addition of Trition-X100 could steady the major conformation and show good HSQC (Heteronuclear Single Quantum Coherence) spectrum (Supplementary Figure S1 a and b). The solution structure of TritonX-100 ligand HaSCP-2 was derived from 2262 distance constraints and 219 angle constraints from chemical shift analysis. 100 refined structures were generated and the best 20 conformers, those with lowest energy that showed the fewest constraint violations, were chosen to represent the solution structure of HaSCP-2. The lowest energy conformer was chosen for further analysis. The structural statistics are summarized in Table 1.

Similar to the SCP-2 of human, rabbit and mosquito (Fig. 3C), *H. armigera* SCP-2 exhibits a typical α/β fold arrangement in secondary structure. The fold is a common characteristic that the sterol carrier protein superfamily possesses as defined by structural classification of proteins^25^. The order of secondary structural elements in HaSCP-2 is as follows: α1-α2-βIII-βII-α4-α3-α5-βIV-βI. The HaSCP-2 fold is dominated by a four-stranded β-sheet that exhibits strand order of 3-2-4-1 with a layer of five α-helices that cover the β-sheet in the three-dimensional structure. The folds of α-helices and β-sheets interact together to produce a large cavity that creates a hydrophobic pocket for accommodating small molecules such as sterols and lipids. The program DALI^38^ was used to search the Protein Data Bank for proteins with structures similar to HaSCP-2. Results showed that nine structures identified belong to SCP-2 family with sequence identify 20–47% and r.m.s.d. 2.0–3.3 Å (Table 2). And it was found that the most similar structure is that of Oryctolagus cuniculus (European rabbit) (PDB code 1c44, sequence identity was 47%; r.m.s.d. was 2.0 Å). The SCP-2 from Homo sapiens HsMEF-2 (PDB code 1ikt, sequence identity was 30%; r.m.s.d. was 2.6 Å) and that from *Aedes aegypti* (PDB code 1pz4, sequence identity was 20%; r.m.s.d. was 2.4 Å) were also similar. These results were correspondent to the sequence alignment analysis showed in Fig. 2. The major structural difference between HaSCP-2 and AeSCP-2 was that there was no loop between α-helix 1 and β-sheet I, which resulted in the different opening of cavity between HaSCP-2 and AeSCP-2. In HaSCP-2, one opening of the cavity was lying near α-helix 1, which is similar to that of HsMFE-2.

**Figure 4:**
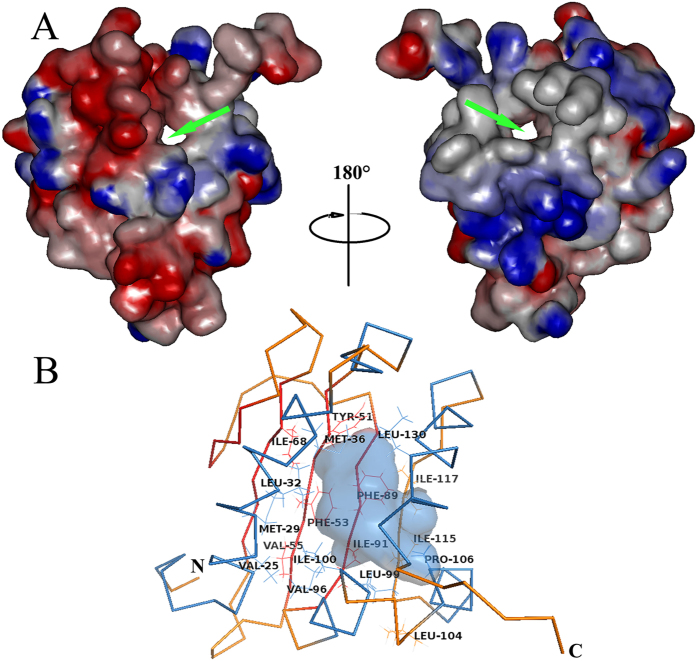
Electrostatic surfaces potential of HaSCP-2 and the hydrophobicity of ligand-binding cavity. (**A**) Negative surface potentials are in red, positive surface potentials are in blue and neutral surface potentials are in white. The orientation of SCP-2 in left is corresponding to that in Fig. 4. This side of the molecule is referred to as the front-side and the orientation of SCP-2 in right was obtained by a 180° clockwise rotation about a vertical axis in the plane of the paper. The green arrows showed the proposed tunnel with putative entrance and exit. (**B**) the 18 hydrophobic amino acids within the interior surface of the ligand-binding cavity found by siteID Find Pocket program in SYBYL software.

The three-dimensional structure of HaSCP-2 (Fig. 4A) shows the hydrophobic cavity to possess two putative openings which could function as either an effective entrance or exit of the transporting tunnel, which was found in the X-ray crystal structure of human SCP-2 (MFE-2)^32^. Careful inspection of HaSCP-2 tertiary structures reveals that one opening is surrounded by α1, α2, α3 and α5 helices folds, while the other is by α4, α5 and stranded β-sheets. Therefore, the tunnel is structurally well-suited for lipids and sterol molecules to enter the cavity and subsequently bind to HaSCP-2. Furthermore, by using siteID Find Pocket program in SYBYL software, the HaSCP-2 three-dimensional structure (Fig. 4B) showed that eighteen hydrophobic amino acids such as V25, M29, L32, M36, Y51, F53, V55, I68, F89, I91, V96, L99, I100, L104, P106, I115, I117 and L130 stayed in the inner surface of the cavity, which are likely serving as lipid and/or sterol interaction sites. Besides, two hydrophilic amino acids T128 and Q131, which has been considered as sterol interaction sites^30^, were lying adjacent to the opening of the cavity.

### Docking simulation

**Figure 5:**
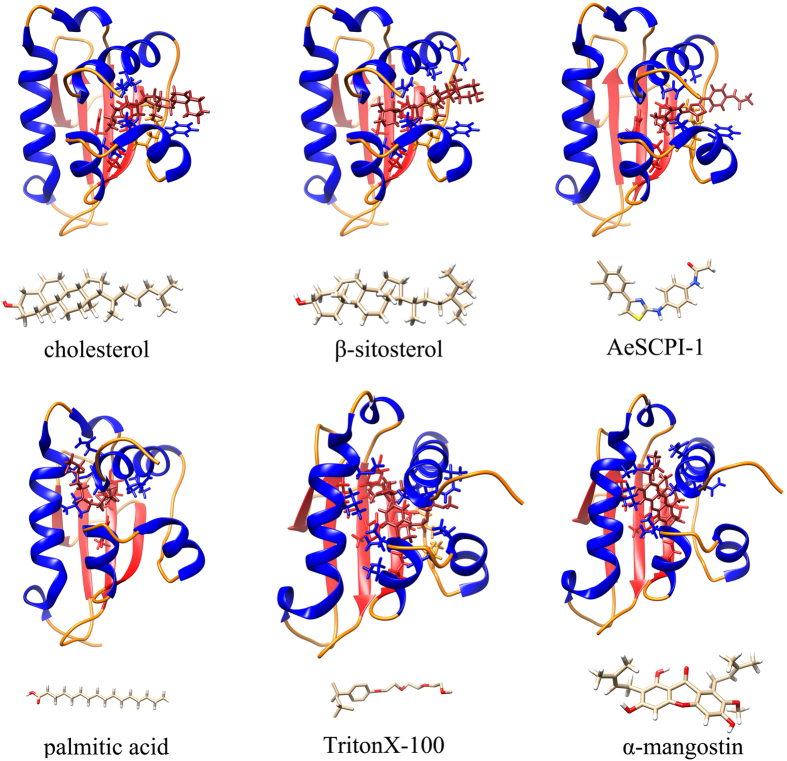
The structures of HaSCP-2 complexed with different ligands (including inhibitors) by the docking simulations. The analysis of molecular docking was carried out by using SYBYL-7.3 program package (Tripos International, St. Louis, MO, USA). The amino acid residues that interacted with ligands were searched by UCSF chimera 1.8.1 software.

In order to study the interaction of HaSCP-2 with lipid and sterol molecules, we carried out the docking simulation of HaSCP-2 with cholesterol, β-sitosterol, palmitic acid, and TritonX-100. The molecular structures of these four ligands were drawn by ChemOffice Software. The structural conformation with the lowest energy of each ligand was chosen to perform molecular docking with HaSCP-2 by using SURFLEX module of SYBYL-7.3 program package (Fig. 5). The docking results indicated that lipid such as cholesterol, β-sitosterol and palmitic acid could bind to HaSCP-2 with high docking scores (Table 3). The prediction of the potential binding sites on HaSCP-2 was conducted during the docking simulations. The predicted binding site residues for the ligands are shown in Table 4. It is implied that HaSCP-2 may interact with cholesterol or fatty acids through these important sites.

It was found that in the yellow fever mosquito (*Aedes aegypti*)^39^, the tobacco hornworm (*Manduca sexta*)^12^ and the cotton bollworm (results described in the following text) that SCP-2 inhibitors (SCPIs) were able to inhibit the binding of cholesterol to SCP-2 by competitive binding to HaSCP-2, which effectively affects the cholesterol uptake and metabolism in insects. Aim to clarify the competitive binding of SCPIs to HaSCP-2, the interaction of two inhibitors, AeSCPI-1(N-(4-{[4-(3,4-dichlorophenyl)-1,3-thiazol-2-yl]amino}phenyl) acetamide hydrobromide) and mangostin (1,3,6-trihydroxy-7-methoxy-2,8-bis(3-methylbut-2-enyl)-9H-xanthen-9-one), with HaSCP-2 was analyzed by molecular docking simulations in the same manner. The results demonstrated that SCPIs bound with HaSCP-2 (Table 3 and Fig. 5). The potential binding sites were shown in Table 3. The results of the SCPI docking simulation revealed that these hydrophobic amino acids, which are putative competitive SCPI and mangostin binding sites, are likely also the key sites of HaSCP-2 binding with cholesterol, and are crucial for cholesterol absorption and transportation in the cotton bollworm (Table 4). Further analysis of these important functional binding sites may lead to new potential targets for pesticides.

### Ligand binding activity of HaSCP-2 protein

**Figure 6:**
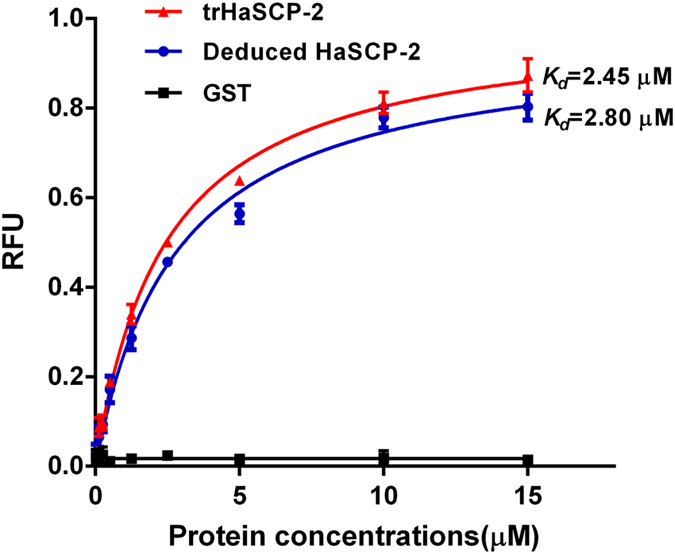
NBD-cholesterol binding assay of HaSCP-2. Both full length protein and truncated protein (trHaSCP-2) were used for cholesterol binding assay. The net changes in NBD-cholesterol fluorescence in intensity (RFU = Relative fluorescence unit) was shown. The protein concentration was from 0.025 μM to15 μM and the final concentration of NBD-cholesterol for each assay was 0.25 μM. GST was used as the negative control. The dates were processed using GraphPad PRISM4.0 software.

NBD-cholesterol binding assays were performed to investigate the biological function of HaSCP-2 in this study. Both the recombinant full length and trHaSCP-2 were examined for NBD-cholesterol binding (*K*_*d*_). Glutathione S-transferase (GST) was used as a negative control as before^12^. The results were shown in Fig. 6. The final concentration of NBD-cholesterol was kept constant at 0.25 μM and the concentration of full length or truncated HaSCP-2 was tested in a range from 25 nM to 15 μM. The increasing concentration of full length or truncated HaSCP-2 led to the increase in the fluorescence emission value of NBD-cholesterol. The GST negative control exhibited no change in the fluorescence emission value (Fig. 6). The binding curves were analyzed by using the one site binding non-linear regression model adopted in the similar studies. It was found that both full length and truncated HaSCP-2 showed high affinities to cholesterol, with the *K*_*d*_ of truncated HaSCP-2 being 2.45 μM (R^2^ = 0.99) and *K*_*d*_ of full length HaSCP-2 being 2.80 μM (R^2^ = 0.99) (Fig. 6). As expected, the GST negative control showed little binding affinity to NBD-cholesterol. It is quite likely that the N-terminal and C-terminal residues that were deleted may not be necessary for the ligand binding activity of HaSCP-2, as shown that no significant differences in binding affinity between the truncated HaSCP-2 and full length HaSCP-2. These bioassay results demonstrated that HaSCP-2 may play an important role in the cholesterol absorption and transportation in the cotton bollworm.

### Mutagenesis of HaSCP-2 and the effects of ligand binding

**Figure 7:**
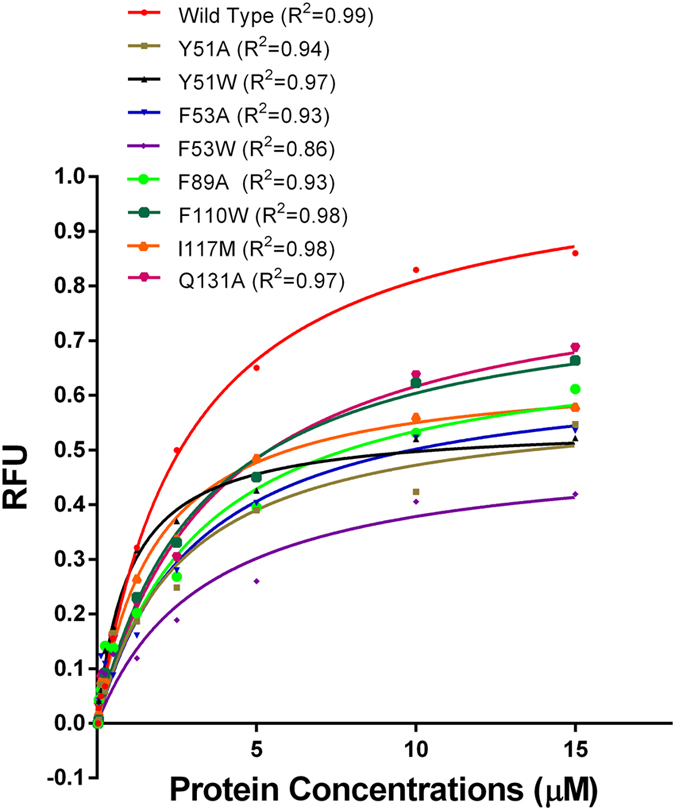
The NBD-cholesterol binding assay of wild type and mutant HaSCP-2s. The net changes in NBD-cholesterol fluorescence in intensity (RFU = Relative fluorescence unit) was showed with the concentration of NBD-cholesterol was 0.25 μM and in the presence of increasing concentrations of each protein (0.025 to 15 μM). The background NBD-cholesterol fluorescence was deducted from each assay. The data from three replicates for each assay was processed using GraphPad PRISM4.0 (single binding site and nonlinear regression model). R^2^ values of each non-linear regression curve were shown.

In order to further confirm the key residues of HaSCP-2 that are involved in the ligand binding, based on the HaSCP-2 structure and docking simulation results we selected several predicted amino acid residues as shown in Table 3 (cholesterol and β-sitosterol) to perform single site mutation experiments to investigate the mechanism of ligand binding in HaSCP-2. The point mutations in HaSCP-2 that caused reductions in binding of NBD-cholesterol were shown in Fig. 7. As shown in Fig. 7, mutations of Y51A, Y51W, F53A, F53W, F89A, F110W, I117M and Q131A in HaSCP-2 reduced the ability of the mutants to bind with NBD-cholesterol compared with wild type (Fig. 7 and Table 5). All of the mutations except Y51W caused significant change in the binding affinity compared to the wild type (Fig. 7 and Table 5), which suggested that these amino acids may be the important functional sites involved in the intracellular hydrophobic interaction between HaSCP-2 and cholesterol. This was consistent with the mutation analysis in AeSCP-2 and SlSCP-2^30^,^31^. Especially, the mutation of F53W caused more decreasing in binding affinity than in the other mutations (Fig. 7), which indicated that F53 was an important site involved in the hydrophobic interaction of HaSCP-2 with cholesterol and may be more critical for the ligand binding ability of HaSCP-2 than other sites. This is consistent with the finding in AeSCP-2 with the F32 (corresponding to F53 in HaSCP-2) mutation in mosquito and F53 mutation in SlSCP-2 site^30^,^31^. Different from AeSCP-2, Y51A mutations decreased the binding affinity of SCP-2 to NBD-cholesterol (Fig. 7). Because there is just a Gly residue between Y51 and F53 site in HaSCP-2 which is different from AeSCP-2, it was speculated that the mutation of Y51 may easily affect the interaction between F53 and Y51 and consequently resulting in the decreasing of ligand binding affinity. This further suggested that F53 was an important site, which was considered to be interacted with fatty acid and TritonX-100^30^,^31^. It was the first time to find that I117M mutation could decrease the binding affinity to NBD-cholesterol. The reason was that I117 amino acid was mutated to a weaker hydrophobic amino acid M, which reduced the hydrophobicity of the cavity. Although molecular docking conducted that I91 was an important site for ligand binding, in fact the I91M mutant protein couldn’t be obtained due to the poor solubility of the mutant(data not shown), which suggested that I91 may be an important sites to keep the structural configuration of HaSCP-2.

### SCPIs competition binding with HaSCP-2 and effects of SCPIs on cholesterol absorption *in vivo*

**Figure 8:**
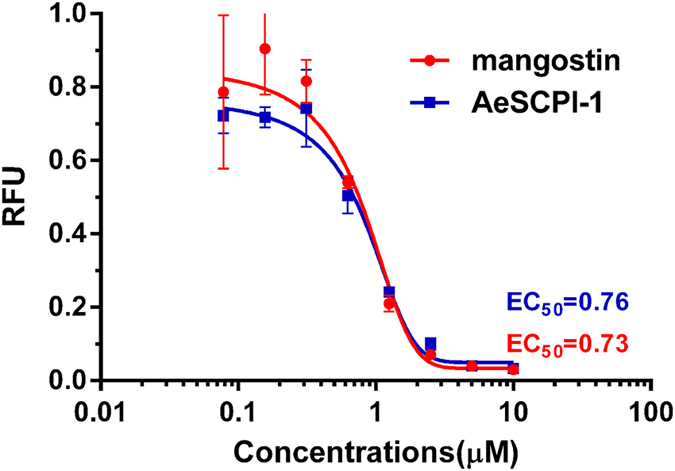
NBD-cholesterol and SCPIs competition binding assays to HaSCP-2. Assays for NBD-cholesterol and sterol carrier protein-2 inhibitors (SCPIs; AeSCPI-1 and mangostin) competitive binding to HaSCP-2. The background NBD-cholesterol fluorescence (NBD-cholesterol alone in the reaction buffer) was deducted from each assay. Shown are net changes in NBD-cholesterol fluorescence in intensity (RFU = Relative fluorescence unit) in the presence of increasing concentrations of each SCPI (0.08 to 10 μM). The data were processed using GraphPad PRISM4.0 software.

In previous studies, it was found that SCPIs caused mortality in the cotton bollworm larvae at early stages^17^. In order to verify the inhibitory effect of SCPIs on cholesterol binding, NBD-cholesterol competition assays were carried out using AeSCPI-1 and mangostin. AeSCPI-1 and mangostin inhibited the NBD-cholesterol binding to HaSCP-2 in a concentration dependent manner (Fig. 8). The 50% maximal effective concentration (EC_50_) of SCPI-1 was 0.76 μM (R^2^ = 0.99), and that of mangostin is 0.73 μM (R^2^ = 0.97). These results indicated that SCP-2 inhibitors had high binding affinity to HaSCP-2 and were able to compete with cholesterol for binding to HaSCP-2.

**Figure 9:**
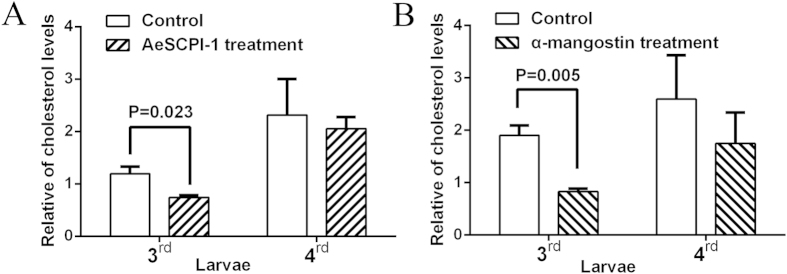
The levels of cholesterol in *Helicoverpa armigera* larvae treated by SCP-2 inhibitors. The cholesterol levels in the fat body of *Helicoverpa armigera larvae* which were treated with two inhibitors (AeSCPI-1 and mangostin) were determined. (**A**) For AeSCPI-1 treatment. Larvae were treated with AeSCPI-1 at day 1 3rd and day 1 4th instar stage, respectively. The fat body samples from 5th instar larvae (3 larvae/sample) were taken at 72 hour post 5th molt. The concentration of cholesterol and total protein from each sample were measured. Mean and standard deviation are shown (N = 3). (**B**) For mangostin treatment. Larvae were treated and samples were measured in the same way as AeSCPI-1. The data were processed using GraphPad PRISM4.0 software. Relative cholesterol levels were defined as: cholesterol concentration (mg/ml)/protein (mg).

To investigate the effects of SCPI suppression on cholesterol absorption and accumulation in the fat body*, in vivo* experiments were conducted. The inhibitory effects of SCPIs on cholesterol uptake were tested at the early stages of 3rd and 4th instar larvae. *H. armigera* larvae were treated with SCPI-1 and mangostin. Because the fat body of insects is the main tissues of cholesterol storage, the fat bodies of the treated larvae were dissected at 5th instar and the concentration of cholesterol in the fat body was measured. The results showed that for AeSCPI-1 treatment, the cholesterol levels was significantly lower than that of the control group when the larvae were treated at 3rd instar stage (*p* = 0.023 Fig. 9A 3rd). Similarly, for the mangostin treatment group, there was a 2-fold decrease in the cholesterol levels (*p* = 0.05) (Fig. 9B 3rd) when the treatment started at 3rd instar stadium. All the result together indicated that SCP-2 inhibitors could significantly affect the cholesterol absorption in the young larvae, leading to the lethal effects on larvae. However, for the inhibitor treatment starting at the 4th instar stage, there were no significant differences in the levels of cholesterol accumulation between the treatments and controls (Fig. 9A 4th and 9B 4th). This bioassay experiment suggested that inhibitor treatment starting at late larval stage probably had less impact on the cholesterol uptake than that at early larval stages. These results were consistent with our previous observations that the young larvae were more sensitive to SCP-2 inhibitors than old ones and the inhibition effect of SCPIs in young larvae could cause severe biological defects in the insect^17^.

## Discussion

The NMR structure of a lepidopteran insect sterol carrier protein-2 was determined for the first time in this study. By comparison of the structures of the SCP-2 family members, including HaSCP-2 in this study, and human SCP-2 (1ikt), rabbit SCP-2 (1c44), mosquito SCP-2 (1pz4), a putative SCP-2 from the hyperthermophilic bacterium *Thermus thermophilus* [2cx7 (X-ray) and 1wfr (NMR)], it is found that this protein family has a common α/β-fold arrangement consisting of four to five α-helices and four to five β-strands, implying a high degree of conservation in the structures and functions of vertebrate and invertebrate SCP-2. The functional domain consisting of α-helices and β-strands may be critical for the ligand binding activity of this class of proteins. HaSCP-2 exhibited a hydrophobic channel consisting of five α-helices lying on one side of a plane expanded by stranded β-sheets. The channel is responsible for capturing and transferring small hydrophobic bound molecules such as sterols and lipids. It was noted that the structure of HaSCP-2 is structurally more similar to vertebrate SCP-2 than to dipteran mosquito SCP-2 (Fig. 4). Similar to the reported vertebrate SCP-2, HaSCP-2 consists of five α-helices, whereas mosquito AeSCP-2 structure detected by crystal and NMR methods contains four α-helices with the lack of α-2 helix that exists in vertebrate and HaSCP-2 proteins. This is expected because HaSCP-2 is transcribed from SCP-x/SCP-2 transcript, similar to vertebrates, whereas mosquito SCP-2 is not a fusion of two domains. On the other hand, the HaSCP-2 NMR structure presents a supporting network made up of four stranded β-sheets. This is the same as bacteria *T. thermophiles* SCP-2 NMR structure, but different from human, rabbit and mosquito SCP-2, in which the five stranded β-sheets are found. Furthermore, the structural difference between the dipteran mosquito SCP-2 and the lepidopteran HaSCP-2 may explain the differential specificity of SCP-2s to chemical inhibitors.

It was found that TritonX-100 helped the structure analysis in NMR experiments (Supplementary Figure S1). It is likely that TritonX-100 bound with HaSCP-2 to firm a conformation in the solution. The same interaction between TritonX-100 with SCP-2 domain was also found in human multifunctional enzyme (PDB 1IKT)^32^. In the X-ray structure determination of human SCP-2 complexed with TritonX-100, Triton X-100, which bears analogy to normal acyl-Co-A substrates of the protein, bound in the hydrophobic tunnel. Thus the positive function of Triton X-100 in SCP-2 structure is definite. Whether or not TritonX-100 was able to enter the hydrophobic tunnel of HaSCP-2 to stabilize the conformation is unclear and needs further studies by labelling TritonX-100 for NMR. Based on the obtained structure data of the SCP-2 superfamily, it was found that human SCP-2 (MsMFE-2), rabbit OcSCP-2, mosquito AeSCP-2 and AeSCP-2L3 function as monomers. Whereas mosquito AeSCP-2 L2, yeast YlSCP-2 and bacterium TtSCP-2 most likely favor for binding fatty acids and form a homodimer^29^,^34^,^35^, HaSCP-2 behaved as a monomeric protein as detected by gel filtration and sedimentation experiments. Thus it is possible that HaSCP-2 executes its function as a monomer within cells.

Using the NMR structure of HaSCP-2 as a template, mutants of HaSCP-2 were created and studied for their ability to interact with ligands. The results showed that a single point mutation could alter the selectivity for the bound ligands, probably by affecting the ligand binding cavity in the protein. Several hydrophobic amino acids inside the hydrophobic pocket of HaSCP-2 were selected to perform mutational analysis and the reduction of in the binding affinity of protein to cholesterol was found. It is possible that the mutation of these residues affect the hydrophobic character of the SCP-2 domain. Whether F53 was mutated to A or W, the binding activity of mutated protein to cholesterol was significantly decreased due to the reduced hydrophobic character of SCP-2 domain. And in comparison, the mutation of F53W caused more decreasing in binding affinity than in the other mutations (Fig. 7), which indicated that F53 may be more critical for the ligand binding ability of HaSCP-2 than other sites. Therefore, F53 is a key site for HaSCP-2 to interact with ligands. This is consistent with the observation in SlSCP-2 and AeSCP-2, in which F53 is correspondent to F32 and Y51 is correspondent to Y37 in AeSCP-2^30^,^31^. Except for F53 site, I117 was identified to be a new important site for the HaSCP-2 hydrophobic interaction with ligands. Overall, we predicted that the mutation on these important sites maybe alter the hydrophobicity of the protein cavity in HaSCP-2, resulting in the reducing accommodation of sterol and lipid molecules. These key functional sites in HaSCP-2 structure would be potential target for insecticides.

Our study describes for the first time the three-dimensional structure of HaSCP-2 by NMR. Docking simulation and mutagenesis clarify the structure-function relationship of HaSCP-2 and reveal the important functional sites of the protein. This study will facilitate the screening of effective environmentally friendly insecticides targeting SCP-2.

## Methods

### The construction of HaSCP-2 recombinant plasmids

Total RNA was isolated from 2 × 10^5^ cultured *Helicoverpa armigera* cells by using Trizol reagent (Invitrogen, Carlsbad, CA, USA), and the first strand cDNA was synthesized using the Reverse Transcription Kit (Invitrogen, Carlsbad, CA, USA). Recombinant HaSCP-2 with a GST-tag was constructed first. Based on the cDNA sequence of the SCP-2 domain of HaSCP-2(GenBank JN582013), we designed two gene specific primers: 5′-gccggatccTACCGCAAAGGATTCGC-3′(forward primer with a BamHI recognition site) and 5′-gccctcgagCTACAGTTTGGAACGAATAGATT-3′ (reverse primer with a Xho I recognition site). The HaSCP-2 gene was amplified by PCR. PCR reaction was performed under the following conditions: 94 °C for 5 min, 35 cycles at 94 °C for 30 s, 61 °C for 30 s, 68 °C for 30 s and a final extension at 68 °C for 5 min. The PCR product was purified and digested with BamHI and Xho I, then inserted into pGEX-KG vector to build the pGEX-KG-HaSCP-2 expression plasmid. Then the expression plasmid was transformed into *Escherichia coli* Top10 cells. Positive clones were sent for sequencing. The recombinant plasmid pGEX-KG -HaSCP-2 was confirmed by nucleotide sequence analysis.

For NMR, the plasmid expressing a truncated HaSCP-2 domain protein was fused with GST-tag (GST/trHaSCP-2. The amino acid residues, MYRKGFADITPRPVAA, at the N-terminus and RSKL residues at C-terminus (Fig. 2) were deleted by designing the gene specific primers to amplify the 17–141 coding region of deduced HaSCP-2 domain. The 17–141 coding region of deduced HaSCP-2 was used as the truncated HaSCP-2 domain. The pair of gene specific primers was as follows: 5′-tttggatccTCCGGCAACCCCGAGGACTTC-3′ (forward primer with a BamHI site) and 5′-gcgctcgagTTAAGATTCGATTCTTCCGGCGGCTT-3′ (reverse primer with a Xho I recognition site). The PCR products encoding the truncated HaSCP-2 domain (trHaSCP-2) were cloned into pGEX-KG vector. The constructed pGEX-KG-trHaSCP-2 plasmid was confirmed by sequencing. The recombinant protein was expressed in *Escherichia coli* (*E. coli*) strain BL21 (DE3) and contained the N-terminal GST tag linked with a thrombin cleavage site.

### The expression and purification of HaSCP-2

*E. coli* BL21 (DE3) strain was grown in 100 ml Luria-broth (LB) media with 100 μg/ml ampicillin at 37 °C for 3 hours until the value of OD_600_ reached 1.0. The *E.coli* cells were induced with 0.2 mM isopropyl-1-thio-D-galactopyranoside (IPTG) and incubated for about 8 hours at 28 °C to maximize the overexpression of the recombinant protein in the cytosolic fraction. The cells were harvested and flash frozen in liquid nitrogen and stored long term at −80 °C.

For the purification of GST/HaSCP-2, the *E.coli* cells were washed in phosphate-buffered saline (PBS: 140 mM NaCl, 10 mM Na_2_HPO_4_, 1.8 mM KH_2_PO_4_, 2.7 mM KCl, pH 7.4) and resuspended in 30 ml of PBS with 5 mM dithiothreitol. The bacterial cells were lysed with ultra-high pressure homogenizer at 1.2 × 10^9^ Pa for 3 times at 4 °C. The cell lysate was centrifuged at 20,000 × g for about 1 hour to remove cellular debris. The fusion protein was purified by GST affinity column (8 ml bed volume, Merck-Novagen KGaA, Darmstadt, Germany) and the GST tag was cleaved by digesting with 250 units of Thrombin (Amersham Pharmacia) on the column at 4 °C overnight. For the following purification, Hitrap QFF 5 ml anion-exchange column (GE Healthcare, Pittsburgh, PA, USA) was used. The protein was washed with PBS buffer at the flow rate of 1 ml/min and the flow-through was collected. HaSCP-2 protein was further purified on Superdex 16/60 200HR gel filtration column (GE Healthcare, Pittsburgh, PA, USA) with 50 mM HEPES, 150 mM NaCl, pH 7.5. Purified protein was then lyophilized and stored at −80 °C.

### NMR spectroscopy

For NMR spectroscopy, uniformly isotope-labeled HaSCP-2 domain was prepared by culturing the bacteria in M9 minimal medium using ^15^NH_4_Cl (for U-^15^N-labeled protein) as the sole nitrogen source or ^15^NH_4_Cl and ^13^C_6_H_12_O_6_ (for U-^13^C, ^15^N-labeled protein) (Cambridge Isotope Laboratories Inc.) as the sole nitrogen and carbon sources, respectively. The concentration of purified protein of the NMR sample was ~1.0 mM, dissolved in 20 mM pH 6.5 phosphate buffer containing 120 mM NaCl, 2.5 mM Triton-X100 and 0.02% NaN_3_. All NMR spectra were acquired at 298 K on Agilent DD2 600 MHz spectrometer equipped with a cold probe. The sequential backbone resonance assignments were achieved by using standard triple-resonance experiments: HNCACB^40^, CBCA(CO)NH^41^ and HNCO^42^. Non-exchangeable side chain resonance assignments were carried out with the help of the following spectra: HBHA(CO)NH^43^, C(CO)NH^44^, HCCH-TOCSY^45^ and 13C-edited NOESY^46^. Inter-proton distance restraints were derived from three NOESY spectra (all with 100 ms mixing time): 15N-edited NOESY and 13C-edited NOESY for the respective aliphatic and aromatic regions. The spectra were processed with the program of NMRPipe^47^ and analyzed with CcpNmr program suite^48^. Structure calculation and NOE assignment were performed simultaneously by using the program CNS^49^ and ARIA2^50^ which has a portal to the CcpNmr suite. Hydrogen bonding restraints were generated from the standard secondary structure of the protein based on the NOE patterns and backbone secondary chemical shifts. Backbone dihedral angle restraints (ϕ and ψ angles) were derived using the program DANGLE^51^ incorporated in the CcpNmr suite. A total of 200 structures were calculated and the 20 structures with the lowest total energy and least experimental violations were selected to perform a refinement procedure in water. The refined structure ensemble was selected to represent the HaSCP-2 structure. The atomic coordinates of the HaSCP-2 has been deposited in the Protein Data Bank with the accession code of 4UEI and the chemical shift assignments of the domain have been deposited in the Biological Magnetic Resonance Data Bank with accession number of 111555 (http://www.bmrb.wisc.edu). The protein structure ensemble was displayed and analyzed with the software of MolMol^52^ and UCSF chimera 1.8.1^53^.

### Docking simulation analysis

In order to detect the binding site of HaSCP-2 with ligands, which help to illustrate the accurate binding model and the mechanism of interaction, molecular docking analysis were carried out by using SYBYL-7.3 program package (Tripos International, St. Louis, MO, USA). Before docking simulations, the structures of ligands (cholesterol, β-sitosterol, palmitic acid) were drawn by using ChemOffice 2008 (CambridgeSoft) and the minimum energy conformation of each ligand were processed by MM2 program^54^. TritonX-100 ligand was separated from PDB 1IKT. For docking experiment, the protocol was generated by keeping the default parameters (mode of Automatic, threshold factor of 0.1 Å and a bloat of 0 Å). The ligands were then prepared by protocol of Surflex_for_searching. The Docking Mode of Surflex-Dock GeomX was utilized to generate 20 conformations per structure and the conformation of maximum total score was chosen for the further analysis. To found the contact residues between protein and ligands, UCSF chimera 1.8.1 was used to find atoms with van der Waals (VDW) overlap ≥−0.4 angstroms.

### Mutation

Single site amino acid mutations in the pGEX-KG/trHaSCP-2 were introduced using the QuikChange II Site-Directed Mutagenesis kit (Stratagene, La Jolla, CA). The Y51, F53, F89, I91, F110, I117 and Q131 sites of HaSCP-2 were selected to perform mutation. Mutant plasmids were sequenced to confirm the introduced point mutations.

### Ligand and SCPIs binding with HaSCP-2

HaSCP-2 binding activity to cholesterol was determined by using NBD-cholesterol binding assays. In truncation experiments, deduced HaSCP-2 and truncated HaSCP-2 were used for NBD-cholesterol binding assay; while as in mutation experiment, truncated HaSCP-2 (defined as wild type HaSCP-2) and mutated HaSCP-2 were assayed. HaSCP-2 was diluted in 10 mM KHPO_4_ buffer (pH 7.4) to a serial of varied concentrations (1 nM-10 μM), respectively. 50 μl of each concentration of HaSCP-2 solution (either wild type HaSCP-2 or mutated HaSCP-2) was mixed with 50 μl of NBD-cholesterol solution containing 1 μM NBD-cholesterol in 10 mM KHPO_4_ buffer (pH 7.4). And the total 100 μl of mixture was incubated at room temperature for 5 minutes. Then, the samples were applied to measure the fluorescence signals using a fluorescence microplate reader (GeminiXPS, Molecular Devices). Fluorescence was excited at 485 nm and the emission was measured at 530 nm. Date was processed using a simple, single binding site, non-linear regression model in GraphPad PRISM® software version 4.0 (GraphPad Software Inc., San Diego, CA). The equation we used is equation 1:


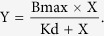


The binding affinity is defined as *K*_*d*_. In the equation, X is concentration of NBD-cholesterol, Y is the total binding defined by fluorescent intensity unit and B*max* is the maximum specific binding to be fit. GST was used as a negative control. Binding assays for the control was carried out by the same way described above for HaSCP-2 protein.

Aim to evaluate the SCP-2 inhibitors (SCPIs) inhibitory effects on cholesterol binding to HaSCP-2, the NBD-cholesterol competition assays was performed with two kinds of SCPIs (SCPI-1 and mangostin), respectively. In the presence of each SCPI with an increasing concentrations (0–10 μM), HaSCP-2 (with final concentration of 5 μM) was incubated with NBD-cholesterol (with final concentration of 1.25 μM) in 100 μl of 10 mM KHPO_4_ buffer (pH 7.5) at 25 °C. Mixture samples were added into 96-well plates and fluorescence intensity was measured on the microplate reader (GeminiXPS, Molecular Devices). Excitation was at 485 nm and emission was measured at 530 nm. The results are analyzed from three repeated experiments with standard deviations. All the data were processed using a single site competition, non-linear regression model in the GraphPad PRISM software version 4.0 (GraphPad). The 50% effective concentration (EC50) was obtained using the equation 2:


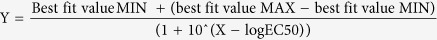


### Biological assays in *H.armigera lavae*

The *H.armigera* larvae were reared according to previous methods^17^. Larvae were reared at 26 °C in 75–80% humidity and a photoperiod of 14 h light and 10 h dark. The *H.armigera* larvae were treated with two inhibitors, SCPI-1 and α-mangostin in the bioassay. Both inhibitors were dissolved in dimethyl sulfoxide (DMSO) at a concentration of 200 mM. Then SCPIs were diluted with 95% ethanol to final concentrations of 3.75 mM for SCPI-1 and 12.5 mM for mangostin. SCP inhibitors treatments were carried out at third instar and forth instar, respectively. The day 1 third and day 1 fourth instar larvae were placed on a block of 2 g diet foods containing 40 μl of each inhibitor with defined final concentration above. Fresh diets were provided every other day. The larvae were grown to fifth instar stadium. For two different stages (3rd or 4th instar) treatments, 3 larvae/each sample from 3-day-old fifth stage were picked up, respectively and the fat body tissues were dissected out for cholesterol concentration measurements. Because the fat body of insects is the main tissue of cholesterol storage, the fat bodies of the treated larvae were dissected at 5th instar and the concentration of cholesterol in the fat body was measured. Non-treated wild type *H.armigera* was used as a negative control. The concentration of cholesterol was assayed using the tissue total cholesterol assay kit (Applygen Technologies Inc., Beijing, China) and the total protein of each sample was determined by BCA protein assay kit (Thermo Scientific Pierce, Rockford, IL, USA). Relative concentration of cholesterol was defined as: cholesterol concentration (mg/ml)/total protein (mg).

### Statistical analysis

By using the GraphPad PRISM software version 4.0 (GraphPad), data were analyzed with two-way ANOVA (GLM procedure) to determine if several components of the biological parameter in the control groups and treated groups differed significantly. Unpaired t-test with Welch’s correction was performed for two groups with unequal numbers of samples to determine whether the differences were significant using the GraphPad PRISM software version 4.0 (GraphPad)^55^.

## Additional Information

**How to cite this article**: Ma, H. *et al.* NMR structure and function of *Helicoverpa armigera* sterol carrier protein-2, an important insecticidal target from the cotton bollworm. *Sci. Rep.* **5**, 18186; doi: 10.1038/srep18186 (2015).

## Supplementary Material

Supplementary Information

## Figures and Tables

**Table 1:** Structural statistics for the family of 20 structures of HaSCP-2 in aqueous solution.

Unambiguous distance restraints	
Intraresidue (i-j = 0)	930
Sequential (|i-j| = 1)	415
Medium range (2 ≤ |i-j| ≤ 4)	227
Long range (|i-j| ≥ 5)	386
Hydrogen bonds	29
Ambiguous distance restraints	275
Total	2262
Dihedral angle restraints	
Φ	109
Ψ	110
Total	219
Mean r.m.s. deviations from the experimental restraints	
Distance (Å)	0.30 ± 0.0015
Dihedral angle (^o^)	0.53 ± 0.10
Mean r.m.s. deviations from idealized covalent geometry	
Bond (Å)	0.0038 ± 0.00012
Angle (^o^)	0.54 ± 0.015
Improper (^o^)	1.40 ± 0.072
Ramachandran plot^a^	
Well-ordered residues^b^ in HaSCP-2% residues in the most favorable regions	90.8
additional allowed regions	8.6
generously allowed regions	0.7
Disallowed regions	0.0
Atomic r.m.s. differences (Å)	
Residues 19–128	
Backbone heavy atoms (N, Cα, and C’)	0.398
Heavy atoms	0.840

None of the structures exhibits distance violations greater than 0.3 Å or dihedral angle violations greater than 4˚.

^a^The program PROCHECK (http://www.biochem.ucl.ac.uk/~roman/procheck_nmr/procheck_nmr.html) was used to assess the overall quality of the structures.

^b^Residues 18–79,82–117,120–134.

**Table 2:** Proteins with similar structures to HaSCP-2^a^.

Organisms	PDB	RMSD^b^	%id^c^	Experiment	Description^d^
*Oryctolagus cuniculus*	1c44	2	47	X-ray	SCP-2
*Homo sapiens*	2c0l-B	3.3	42	X-ray	SCP-2
*Homo sapiens*	1ikt	2.6	30	X-ray	MFE-2
Homo sapiens	1qnd	3.2	36	NMR	SCP-2
*Aedes Aegypti*	2qzt	2.3	25	X-ray	SCP-2L2
*Aedes Aegypti*	3bkr	2.7	20	NMR	SCP-2L3
*Aedes Aegypti*	1pz4	2.4	20	X-ray	SCP-2
*Aedes Aegypti*	2ksh	2.4	19	NMR	SCP-2
*Aedes Aegypti*	2ksi	2.3	18	NMR	SCP-2
*Yarrowia lipolytica*	4jgx	2.2	24	X-ray	SCP-2
*Archaeoglobus fulgidus*	3bn8	2.3	18	X-ray	SCP-2
*Thermus thermophilus*	2cx7	3.2	16	X-ray	SCP-2

^a^Searching proteins with structures similar to HaSCP-2 from Protein Structure Database by DaliLite v. 3 program (http://ekhidna.biocenter.helsinki.fi/dali_server/start).

^b^RMSD (The root-mean-square deviation) is the measure of the average distance between the backbone C_α_atoms of superimposed proteins. The unit is Å.

^c^%id mean the % of sequence identity.

^d^SCP-2 means reported sterol carrier protein-2 or putative sterol carrier protein-2; MFE: the abbreviate of peroxisomal multifunctional enzyme type 2.

**Table 3:** Molecular docking analysis of HaSCP-2 with ligands and SCPIs.

Name of the targets	Total Score	Name of the targets	Total Score
cholesterol	7.26	TritonX-100	8.53
β-sitosterol	7.83	AeSCPI-1	5.64
palmitic acid	7.8	α-mangostin	6.51

**Table 4:** The predicted amino acid residues in the binding pocket of HaSCP-2 by molecular docking.

Name of the targets	Amino acids in the binding pocket
Ligands	
cholesterol	Phe53, Phe89, Ile91, Pro106, Phe110, Leu127, Gln131
β-sitosterol	Phe53, Phe89, Ile91, Leu99, Pro106, Phe110, Ile115, Ile117, Ala124, Met125, Thr128, Gln131
palmitic acid	Leu32, Met36, Tyr51, Phe53, Leu127, Gln131, Gln133, Ala134
TritonX-100	Leu32, Met36, Tyr51, Phe53, Ile68, Phe89, Ile91, Val96, Leu99, Ile100, Pro106, Ile115, Leu127, Thr128, Gln131, Ala134
Inhibitors	
AeSCPI-1	Phe53, Phe89, Ile91, Pro106, Phe110, Ile115, Ile117, Ala124, Gln131
α-mangostin	Leu32, Ala35, Met36, Phe53, Phe89, Ile91, Ile100, Leu127, Gln131, Gln133, Ala134

**Table 5:** Difference between Wild Type HaSCP-2 and Mutants in NBD-Cholesterol binding.

	WT	Y51A	Y51W	F53A	F53W	F89A	F110W	I117M	Q131A
*K*_d_^a^ (μM)	2.78	2.65	1.00	3.07	3.41	3.27	3.24	1.80	3.77
*P* value^b^	−	0.023	0.09	0.031	0.026	0.038	0.019	0.043	0.034

^a^*K*_d_ values were determined using the GraphPad Prism version 4.0 (single-binding site, nonlinear regression model).

^b^*P* values vs. wild type were given for Dunnett’s post T test between the wild type HaSCP-2 and each mutant after a repeated measures analysis of variance.
