# Supplementary material for: NMR structure and function of Helicoverpa armigera sterol carrier protein-2, an important insecticidal target from the cotton bollworm
Source: Sci Rep. 2015 Dec 10;5:18186. doi: 10.1038/srep18186 (PMC4674756; doi:10.1038/srep18186)
Supplement: Supplementary Information [file srep18186-s1.pdf]

NMR structure and function of *Helicoverpa armigera* sterol carrier protein-2, an important insecticidal target from the cotton bollworm

Haihao Ma[1], Yuemin Ma[1], Xuehui Liu[2], David H. Dyer[3a], Pingyong Xu[2], Kaiyu Liu[1], Que Lan[3b], Huazhu Hong[4], Jianxin Peng[1*], Rong Peng[1*]

1 School of Life Sciences, Central China Normal University, Wuhan, P.R.China

2 National Laboratory of Biomacromolecules, Institute of Biophysics, Chinese Academy of Sciences, Beijing, P.R.China

3 Department of Biochemistry[a] and Entomology[b], College of Agricultural and Life Sciences, University of Wisconsin-Madison, Madison, United States of America

4 School of Life Sciences, Wuhan Institute of Bioengineering, Wuhan, P.R.China

[*]Correspondence authors:

Jianxing Peng and Rong Peng

School of Life Sciences

Central China Normal University

No.152 Luoyu Road

Wuhan, Hubei, P.R.China 430079

E-mail: jxpeng@mail.ccnu.edu.cn (Jianxin Peng); prhh@sina.com (Rong Peng)

Telephone: 86-027-67867221

Supplementary Figure

Figure S1. The HSQC spectrum of HaSCP-2 with Triton-X100.

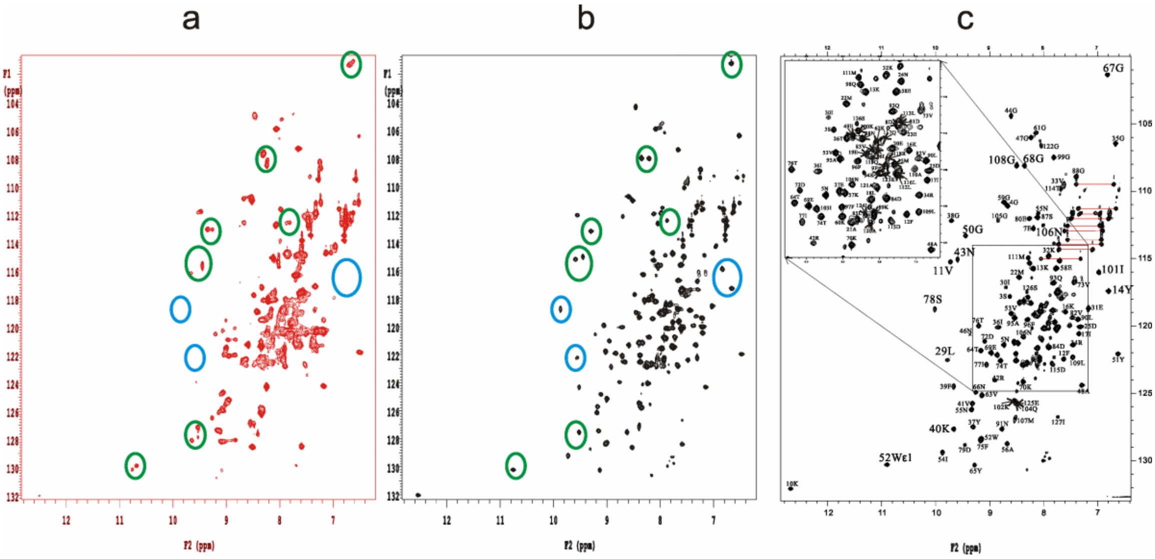

HSQC (Heteronuclear Single Quantum Coherence) spectrums of HaSCP-2 without (a) and with (b) Triton-X100 were shown. (c) indicated assigned HSQC spectrum of HaSCP-2 with Triton-X100 . Green and blue circles in (a) and (b) indicated some of the locations in the spectra showing multi-conformations and the new appeared resonances respectively.
